# Metabolic adaptations in a range‐expanding arthropod

**DOI:** 10.1002/ece3.2350

**Published:** 2016-08-23

**Authors:** Katrien H. P. Van Petegem, David Renault, Robby Stoks, Dries Bonte

**Affiliations:** ^1^ Department of BiologyGhent UniversityGhentBelgium; ^2^ UMR CNRS 6553 EcobioUniversité de Rennes 1Rennes CedexFrance; ^3^ Evolution and ConservationKU LeuvenLeuvenBelgium

**Keywords:** Common garden, essential amino acids, GC‐MS metabolomics, global change, life‐history evolution, *Tetranychus urticae*

## Abstract

Despite an increasing number of studies documenting life‐history evolution during range expansions or shifts, we lack a mechanistic understanding of the underlying physiological processes. In this explorative study, we used a metabolomics approach to study physiological changes associated with the recent range expansion of the two‐spotted spider mite (*Tetranychus urticae*). Mite populations were sampled along a latitudinal gradient from range core to edge and reared under benign common garden conditions for two generations. Using gas chromatography–mass spectrometry, we obtained metabolic population profiles, which showed a gradual differentiation along the latitudinal gradient, indicating (epi)genetic changes in the metabolome in association with range expansion. These changes seemed not related with shifts in the mites’ energetic metabolism, but rather with differential use of amino acids. Particularly, more dispersive northern populations showed lowered concentrations of several essential and nonessential amino acids, suggesting a potential downregulation of metabolic pathways associated with protein synthesis.

## Introduction

During range expansions or range shifts, species’ life histories can evolve on ecological timescales (Phillips et al. 2010). Changing environmental conditions force species to locally adapt, and spatial assortment of dispersive phenotypes leads to increased dispersiveness at the expanding/shifting range edge (Shine et al. 2011). These evolutionary processes of local adaptation and spatial selection affect key life‐history traits such as fecundity, development and dispersal (reviewed in Chuang and Peterson 2016). We therefore expect range‐edge populations to exhibit physiological adaptations that underlie these observed trait evolutions. Such adaptations should be especially significant in energy‐producing pathways, and more particularly in glycolysis (Eanes 2011). Indeed, any elevation of the performance of one life‐history trait augments its energetic and metabolite demands at the expense of other traits (*cfr*. the “Y” model of resource allocation, Van Noordwijk and de Jong 1986; Zera and Harshman 2001), thus modifying the global metabolic network operation. For instance, variations in lipid biosynthesis in association with dispersal strategy highly impact metabolite fluxes through lipid pathways (see Zera 2011 for a review). As a result, life‐history differentiation is expected to be associated with changes in the metabolome (i.e., the set of circulating metabolites within an organism, Oliver et al. 1998), as was, for example, found for aging in *Caenorhabditis elegans* (Fuchs et al. 2010) and reproduction in the malaria mosquito *Anopheles gambiae* (Fuchs et al. 2014).

Metabolomics is a convenient technique which can be used as a candidate approach to explore an organism's response to environmental variations and forthcoming environmental changes (Hines et al. 2007; Miller 2007; Viant 2008; Bundy et al. 2009; Lankadurai et al. 2013; Hidalgo et al. 2014). It provides information on the interaction between an organism's physiology and its natural environment by identifying metabolites of low‐to‐moderate molecular mass within the whole body, cells, tissues, or biofluids. Compared to other –omics technologies such as genomics and transcriptomics, metabolomics has the significant advantage to focus on “downstream” cellular functions (Snart et al. 2015), providing a more direct picture of the functional links between causes and consequences of environmental variation (Foucreau et al. 2012). Essentially, metabolomics can potentially provide a link between genotypes and phenotypes (Fiehn 2002). When applied on individuals originating from different localities from range core to edge, but reared for several generations under common garden conditions, it should provide insights on the physiological adaptations that underlie life‐history evolution during range expansion.

Although a consideration of the whole‐organism physiology allows a better understanding of how life‐history evolution in natural populations might occur and why this evolution is sometimes constrained (Zera and Harshman 2001, Zera 2011; Ricklefs and Wikelski 2002), few studies documented metabolic variation in wild populations along natural gradients (Sardans et al. 2011). Instead, most studies assess plastic or evolutionary responses to environmental stressors by manipulating abiotic variables in controlled environments (Sardans et al. 2011; Colinet et al. 2012; Padfield et al. 2016). Notable exceptions are the studies on *Arabidopsis lyrata* that demonstrated distinct metabolic phenotypes along the species’ latitudinal distribution, with a typical cold‐induced metabolome in the north, indicating adaptation to the local climate (Davey et al. 2008, 2009), but no difference in metabolic fingerprint between large connected versus marginal fragmented populations (Kunin et al. 2009). These studies, however, used plants that were grown from seeds collected directly from the field. Environmental maternal effects can therefore not be excluded.

**Figure 1:**
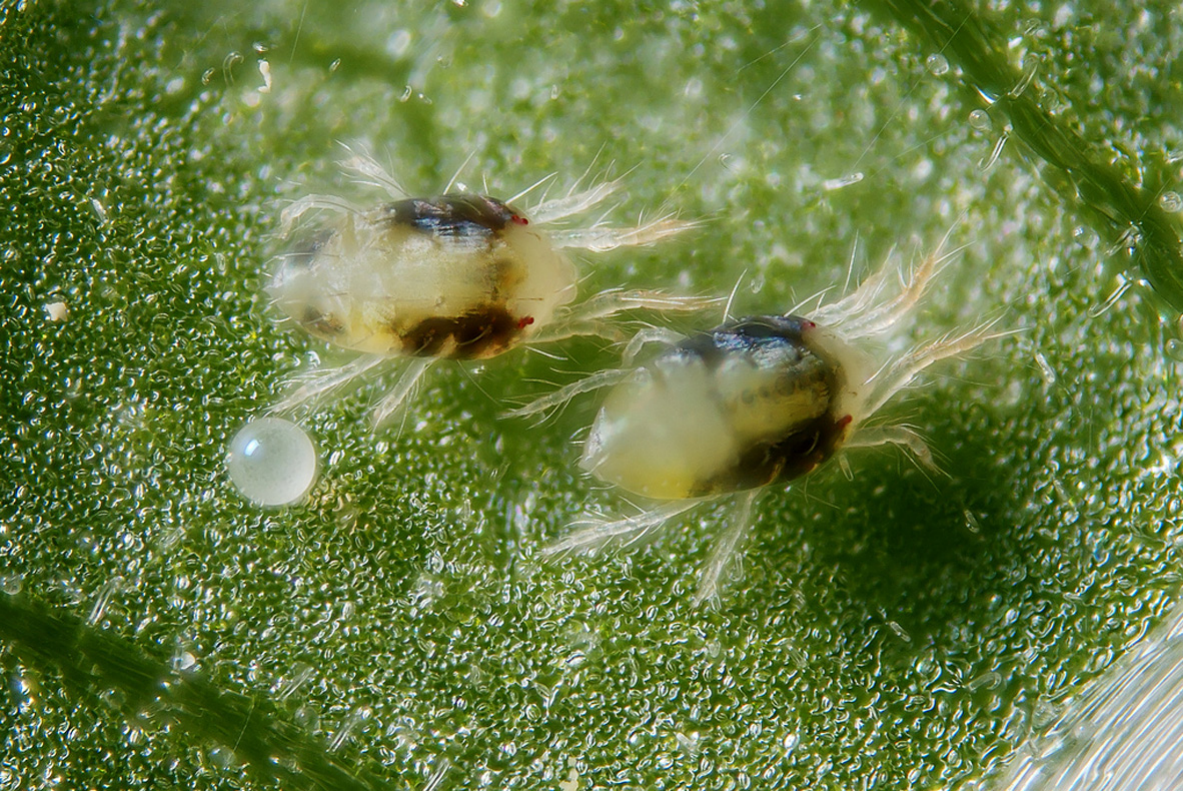
Two females and one egg of the two‐spotted spider mite (*Tetranychus urticae* Koch; Acari, Tetranychidae). This picture was taken by Gilles San Martin (UCL, Belgium), https://www.flickr.com/photos/sanmartin/4883543313/.

The two‐spotted spider mite*, Tetranychus urticae* Koch (Acari, Tetranychidae; Fig. 1), a generalist pest species in greenhouses and orchards, expanded its European range from the Mediterranean to at least southern Scandinavia (K. H. P. Van Petegem, personal observation in 2011) during the last decades (for more information, see Carbonnelle et al. 2007). Previous research with *T. urticae* showed quantitative genetic life‐history differentiation along this latitudinal gradient, with daily fecundity, lifetime fecundity and longevity decreasing from range core to edge, and egg survival, dispersal propensity, and sex ratio increasing from range core to edge (Van Petegem et al. 2016). We expected this life‐history differentiation to be associated with an evolution of the species’ intermediary metabolism, which would manifest into distinct metabolic phenotypes among populations sampled along its expansion gradient.

Using a metabolomics approach, this study aimed to test (1) whether the metabolome of *T. urticae* evolved during its recent range expansion; that is, whether a gradual change in the mite's intermediary metabolism is present from range core to range edge, showing in the appearance of progressively distinct metabolic phenotypes (metabotypes); (2) whether this metabolic differentiation could be associated with the up‐ or downregulation of certain metabolic pathways, for instance enhanced glycolytic activities or lipid metabolism; and (3) whether this evolutionary change in the species’ metabotype is associated with the life‐history differentiation that has occurred during its range expansion.

## Materials and Methods

### Field sampling and common garden

**Figure 2:**
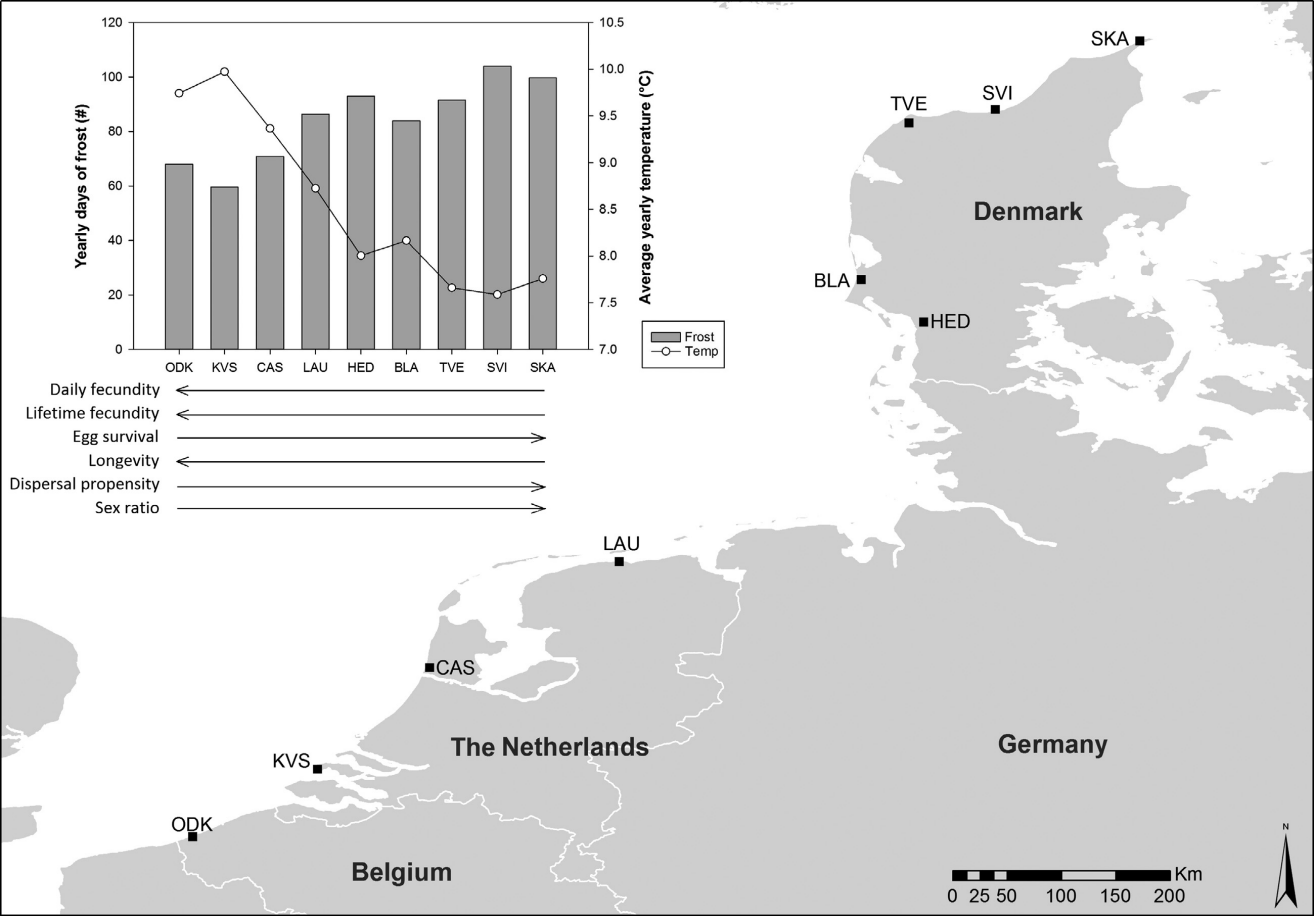
The map shows the nine field collection sites, which are situated in Belgium, the Netherlands and Denmark. The graph shows the yearly number of frost days and the average yearly temperature for each collection site along the latitudinal gradient. These climatic data were obtained from FetchClimate (Microsoft Research, Cambridge) and were averaged over a period of 35 years (1980–2015). Below the graph, arrows for each of six life‐history traits depict their trend along the latitudinal gradient (increase and decrease). (For more detailed information, see Appendix S1 and Van Petegem et al. 2016).

In August 2012, we hand‐sampled mites from nine localities (one population per locality) along an 800‐km latitudinal gradient from northwestern Belgium to northern Denmark (Fig. 2). Mites were found on infested leaves of *Lonicera periclymenum* (European honeysuckle, five populations) at high latitudes and on *Euonymus europaeus* (European spindle, one population), *Humulus lupulus* (common hop, one population), *Sambucus nigra* (European black elderberry, one population), or *L. periclymenum* (European honeysuckle, one population) at lower latitudes. (More information is provided in Appendix S1). In the laboratory, 50 to several hundreds of mites per population were put on separate whole bean plants (*Phaseolus vulgaris*, variety Prélude – a highly suitable host for *T. urticae*, see Agrawal et al. 2002; Gotoh et al. 2004) and kept under controlled conditions at room temperature with a light regime of 16:8 LD. After one generation, 10 adult female mites per population were taken from their bean plant and put on a piece of bean leaf (±30 cm^2^) on wet cotton in a petri dish. Two such petri dishes were prepared for each population. The petri dishes were then used to create a pool of synchronized 2‐day adult female mites for each population (2‐day adult females were preferred, as these are significantly bigger than fresh adults). For this purpose, all females were allowed to lay eggs during 24 h in a climate room at 27°C (an optimal temperature for our study species, see Sabelis 1981), with a light regime of 16:8 LD. The resulting same‐aged eggs were subsequently left to develop until they were 2‐day adult mites, of which only females (which are easily visually recognized) were selected. As mites were kept in common garden for two generations, all direct environmental effects were excluded. Furthermore, as the common garden conditions were optimal for *T. urticae* (bean as host plant, 27°C, 16:8 LD and relatively low mite densities), we could reasonably assume that potential differences among metabotypes of mites from different origins (populations) did not result from differential responses to restrictive/stressful rearing.

### Metabolomic profiling using gas chromatography–mass spectrometry

As we wanted to scan metabolites from different metabolite families (because of their various but connected roles in general organismal physiology), we used gas chromatography–mass spectrometry (GC‐MS) metabolomics (Koek et al. 2011; Khodayari et al. 2013). For each population, we constructed the metabolomic profile of five replicated pooled sets of 50 2‐day‐adult female mites (four for locality “SKA,” where one set was lost). Each set was placed in a microtube and directly transferred to −80°C. To be able to measure true quantities of metabolites, it is important to standardize the initial masses of each extract. However, even when pooling 50 individuals, the masses of the replicates were too low to be accurately measured (the measurement error for the mass of the microtube was greater than the summed mass of the 50 mites). Yet, previous research showed that female adult size does not differ among the nine sampled populations (Van Petegem et al. 2016). We could thus confidently use and interpret metabolite concentrations in nmol per sample. The samples were first homogenized in ice‐cold (−20°C) methanol–chloroform (2:1), using a tungsten‐bead beating equipment (RetschTM MM301; Retsch GmbH, Haan, Germany) at 25 Hz for 1.5 min. After addition of ice‐cold ultrapure water, the samples were centrifuged at 4000 *g* for 5 min at 4°C. The upper aqueous phase was then transferred to new chromatographic glass vials, dried‐out and resuspended in 30 *μ*L of 20 mg·L^−1^ methoxyamine hydrochloride (Sigma‐Aldrich, St. Louis, MO) in pyridine and incubated under automatic orbital shaking at 40°C for 60 min. Subsequently, 30 *μ*L of N‐methyl‐N‐(trimethylsilyl) trifluoroacetamide (MSTFA; Sigma, #394866) was added and the derivatization was conducted at 40°C for 60 min under agitation. The samples were then analyzed in a GC‐MS system (Thermo Fischer Scientific Inc., Waltham, MA), using the same settings as in Khodayari et al. (2013). For this purpose, one microliter of each sample was injected in the GC‐MS system using the split mode (split ratio: 25:1). After that, the selective ion monitoring (SIM) mode (electron energy: −70 eV) was used to search for the 60 primary metabolites that are most often found in arthropod samples and that were included in our spectral database (see Appendix S2 for a complete overview of these 60 metabolites). The SIM mode ensured a precise annotation of the detected peaks. The calibration curves were set using samples consisting of 60 pure reference compounds at concentrations of 1, 2, 5, 10, 20, 50, 100, 200, 500, 750, 1000, 1500, and 2000 *μ*mol·L^−1^. Chromatograms were deconvoluted using XCalibur v2.0.7 software (Thermo Fischer Scientific Inc.). Finally, metabolite concentrations were quantified according to their calibration curves.

### Statistics

A total of 43 metabolites were identified.

In the first step, we examined whether distinct metabotypes existed among the sampled populations of *T. urticae* by running a MANOVA. Nine metabolites were first removed from the dataset because they showed a more than 85% correlation with (an)other metabolite(s). Then, all remaining metabolites were log‐transformed to obtain a normal distribution, which allowed proceeding to the MANOVA.

In the second step, we tested whether the metabolic profile was gradually changing as a function of latitude (from range core to edge) or as a function of one of the six life‐history traits that were previously shown to covary with latitude (daily and lifetime fecundity, egg survival, longevity, dispersal propensity, and sex ratio; see Van Petegem et al. 2016; see also Appendix S3). The concentrations of all 43 metabolites were first autoscaled and transformed (the transformation that best fitted and normalized the data was retained: cube root when looking for covariation with daily fecundity and egg survival; log for latitude; no transformation for lifetime fecundity, longevity, dispersal propensity, and sex ratio). Then, metabolic differences among the nine populations were visualized using partial least squares–discriminant analysis (PLS‐DA). This multivariate analysis was performed using MetaboAnalyst 3.0 (Xia et al. 2009, 2012, 2015). By ordering the populations according to their latitude or according to one of the six life‐history traits covarying with latitude, it was possible to check for trends in the metabolite concentrations. To validate the significance of this interpopulation variation, permutation tests (2000 permutations) were run for replicates (with five replicates – four for SKA – per population) using separation distance (B/W) test statistics. The PLS‐DA provided Variable Importance in Projection (VIP) scores, which gave a first overview of the possible existence of a general pattern in the concentrations of quantified metabolites along our invasion gradient: low VIP scores depict a weak and high scores a strong global pattern. Using a stepwise procedure, only those metabolites with a VIP score of at least 1.2 (1.0 for egg survival because removing the metabolites with a score between 1.0 and 1.2 resulted in a decreased percentage of variation explained) for the first and/or second component were retained for further analysis (compared to 0.8 in Tenenhaus 1998).

In the third step, univariate analyses were performed to test, metabolite by metabolite, whether the global patterns obtained in the previous step could be confirmed. As we aimed at determining whether the metabolite levels showed latitudinal patterns along the expansion, we did not run an ANOVA on individual metabolites, but rather processed regressions. Using SAS 9.4 (2013; SAS Institute Inc., Cary, NC, USA), the linear regressions were run for all influential metabolites (VIP scores >1.2, except for egg survival, as mentioned above). Because all five collected replicates (four for SKA, see earlier) per population originated from only one field sample, we ran the regressions using population averages. As our study is explorative, we wanted to avoid false negatives (with the chance of making a type II error). We therefore did not correct for multiple comparisons (e.g., Bonferroni correction). Given the large number of statistical tests, the limited number of populations, and the fact that we highly smoothed differences among populations by rearing the specimens under common garden conditions for two generations (which is atypical for metabolomics studies, where organisms are usually subject to a stressor to elicit a strong response), such a correction would have greatly diminished the statistical power.

The final step linked the selected individual metabolites with one or more metabolic pathways, thus identifying those pathways that were potentially up‐ or downregulated during the range expansion of *T. urticae*. Pathway enrichment analyses were performed in Metaboanalyst 3.0 (Xia et al. 2009, 2012, 2015) with those metabolites that showed significant effects in the univariate analyses of step three. These pathway analyses were performed with a Fisher's exact test algorithm, which we ran using the metabolic pathways of *Drosophila melanogaster* (no closer relative of *T. urticae* was available in the program, but primary metabolites are anyway highly conserved, especially among nonblood‐feeding arthropods). The algorithm calculates the match (number of hits) between the metabolites in a dataset and the totality of metabolites present in a specific pathway. Furthermore, it uses a pathway topology analysis to compute a value for the impact of these metabolites on the pathway. As multiple comparisons are made, corrected Holm *P*‐values are provided.

## Results

### MANOVA

The metabotypes of the female mites significantly differed among the nine sampled populations (*F*_184,160_ = 2.2, *P* < 0.001). In further analyses, the potential covariation of metabolite levels with latitude or one of the life‐history traits covarying with latitude was then assessed.

### Latitudinal covariation

**Figure 3:**
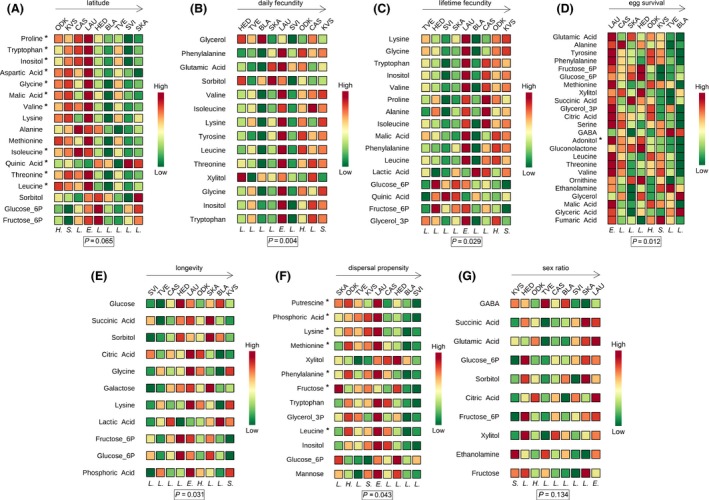
Variable importance plots resulting from the multivariate analyses (PLS‐DA) on the metabolomic data. These plots list those metabolites that, based on their VIP score, contribute the most to explaining the variation among the nine populations in our dataset (ODK, KVS, CAS, LAU, HED, BLA, TVE, SVI, and SKA). The metabolites are ordered from high to low VIP scores for component 1 (an overview off all scores for component 1 and 2 is provided in Appendix S5). The color codes indicate the relative concentration of a given metabolic compound for each population (green = low concentration and red = high concentration). The populations themselves are ordered according to their latitude (A) or from low values at the left to high values at the right for a given life‐history trait (daily [B] or lifetime [C] fecundity, egg survival [D], longevity [E], dispersal propensity [F], or sex ratio [G]). For example, in Figure 3A, LAU is the population with the highest concentration of proline and ODK is the southernmost population (lowest latitude). Below each column (population), a letter signifies the host plant species from which mites were sampled in this population (*H*. = *H. lupulus*,* S. *= *S. nigra*,* L. *= *L. periclymenum*,* E. *= *E. europaeus*). At the bottom of each plot, the *P*‐value resulting from the permutation test is given. At the left side of each plot, an asterisk next to a metabolite name indicates a significant correlation between this metabolite and latitude or the denoted life‐history trait. For example, in Figure 3A, proline shows a significant negative correlation with latitude. (A detailed overview, including *P*‐ and *F*‐values, of all linear regressions is found in Appendix S6).

The PLS‐DA showed a separation among the nine populations, which was visible on 3‐D score plots (see Appendix S4). Of the 43 identified metabolites, 17 had VIP scores of at least 1.2 and were thus retained for further analysis (Fig. 3A). They showed a clear general trend from high values in southern to low values in northern populations (Fig. 3A).

In the subsequent linear regressions, 11 of these 17 metabolite concentrations varied significantly: 10 decreased and one increased with increasing latitude (Fig. 3A and Appendix S6). Among these 11 metabolites, five essential amino acids, three nonessential amino acids (see Rodriguez and Hampton (1966) for an overview of all essential amino acids in *T. urticae* – we defined tryptophan, which is not included in this overview, as essential), and one intermediate of the citric acid cycle can be mentioned.

Pathway analysis indicated that of these 11 metabolites, eight play a significant role in the aminoacyl‐tRNA biosynthesis (total: 67, hits: 8, impact = 0, Holm *P* = 2.7062E‐6) and four in the valine, leucine, and isoleucine biosynthesis (total: 13, hits: 4, impact = 0.9999, Holm *P* = 4.0129E‐4) (both pathway maps are provided in Appendix S7).

### Life‐history covariation

The PLS‐DA showed a separation between the nine (eight for egg survival, for which no data were available for population SVI) populations, which was visible on 3‐D score plots (see Appendix S4). Of the 43 identified metabolites, only those which explained most of the interpopulation variation for a certain life‐history trait (high VIP score) were retained for further analysis. Fourteen were retained for daily and 16 for lifetime fecundity, 20 were retained for egg survival, 11 for longevity, 13 for dispersal propensity, and 10 for sex ratio (Fig. 3B–G). Figure 3 (B–G) shows clear indications of a positive correlation between lifetime fecundity and its 16 selected metabolites. In contrast, Figure 3 (B–G) suggests a negative correlation between the 20 and 13 metabolites selected for, respectively, egg survival and dispersal propensity. For daily fecundity, longevity, and sex ratio, no clear trends were visible.

In the subsequent linear regressions, one significant correlation was found for egg survival (a sugar alcohol) and seven for dispersal propensity (including four essential amino acids and one sugar). No significant results were found for lifetime fecundity, daily fecundity, longevity, and sex ratio (Fig. 3B–G and Appendix S6).

Pathway analysis indicated that four of the seven metabolites which negatively correlated with dispersal propensity play a significant role in the aminoacyl‐tRNA biosynthesis (total: 67, hits: 4, impact = 0, Holm *P* = 0.0417) (the pathway map is provided in Appendix S7). No associated pathways were found for egg survival.

## Discussion

Of the 43 metabolites identified in the GC‐MS analysis, 18 correlated with latitude and/or one or more life‐history traits. More specifically, 11 covaried positively or negatively with latitude, seven showed a negative correlation with dispersal propensity, and one showed a negative correlation with egg survival. Of the 18 different metabolites, 11 amino acids could be shown to play an important role in the aminoacyl‐tRNA biosynthesis and four in the valine, leucine, and isoleucine biosynthesis (see pathway maps provided in Appendix S7).

Contrary to our hypothesis, our results indicate that the life‐history evolution which occurred during the recent range expansion of *T. urticae* (Van Petegem et al. 2016) was not associated with shifts in the mites’ energetic metabolism, but rather with shifts in its anabolism. While our spectral database contained 11 sugars, only one sugar (fructose) accounted for the separation among populations. This suggests that the genes involved in encoding the mite's energetic metabolism (i.e., glycolysis and citric acid cycle, which typically involve sugars) have not been significantly affected during the range expansion of *T. urticae*. Instead, the observed differentiation in the mites’ metabolome probably involved evolutionary changes in the mites’ anabolism, where amino acids play a central role in the metabolic turnover of proteins. In more northern and more dispersive populations, the aminoacyl‐tRNA biosynthesis was downregulated. In this pathway, aminoacyl‐tRNA is formed by charging tRNA with an amino acid. The aminoacyl‐tRNA then serves as a substrate in protein synthesis or plays one of its many other roles in, for example, cell wall formation or antibiotic biosynthesis (Raina and Ibba 2014). In accordance, the valine, leucine, and isoleucine biosynthesis, important for protein synthesis as well (Ahmed and Khan 2006; Tamanna and Mahmood 2014), was downregulated in more northern populations.

The affected amino acids showed decreased concentrations toward higher latitudes and showed a negative correlation with the dispersal propensity of *T. urticae*, which increases toward the north. While, in general, amino acids are considered fundamental for egg production and thus fecundity (Tulisalo 1971; O'Brien et al. 2002; Mevi‐Schutz and Erhardt 2005; Fuchs et al. 2014; but see Heagle et al. 2002), not a single correlation was found for fecundity, despite a clear positive trend in the PLS‐DA. Of the 11 affected amino acids, eight were essential and three nonessential. While the nonessential amino acids could have been synthesised de novo from glucose, the essential amino acids could only have been supplied through the mite's diet (Rodriguez and Hampton 1966). Although all mites were kept in common garden, mites from northern, more dispersive populations were found to contain lower essential amino acid concentrations. In line with the recent finding of Fronhofer and Altermatt (2015) that a dispersal‐foraging trade‐off leads to a reduced exploitation of resources at range margins, our results could indicate that northern, more dispersive mites evolved lower essential amino acid concentrations because they consume less of their food source. We should, however, keep in mind that metabolites were measured only at one point in time and from whole‐organism samples. We are therefore missing the temporal fluctuations of the metabolome over a day, and our data therefore represent only a snapshot of the existing balance in terms of metabolite demand among metabolic pathways.

An important challenge for metabolomics is understanding the relative contribution of environmental and genetic factors in shaping an organism's metabolic phenotype (Bundy et al. 2009). In the current study, mites were kept in common garden for two generations, during which they were reared under optimal, nonstressful conditions. In contrast with most metabolomic studies, mites were thus not subjected to a stressor to elicit a strong environmentally induced plastic stress response. Therefore, any metabolomic differentiation was expected to be far less pronounced compared to stress‐exposure studies. As plastic, environmentally driven field differences among populations were largely levelled out through the two generations in common garden, only genetic factors were retained. Long‐lasting transgenerational plasticity can, however, not be fully excluded and we therefore refer further to (epi)genetic factors (Verhoeven et al. 2016). Although (epi)genetic factors are generally considered less determining than environmental factors (Robinson et al. 2007; Frank et al. 2009; Matsuda et al. 2012), our results demonstrate a clear (epi)genetic signal of metabolic differentiation along *T. urticae*'s invasion gradient. We acknowledge, however, that we cannot exclude neutral processes, such as, for example, serial bottlenecks – including surfing mutations (Klopfstein et al. 2006; Travis et al. 2007) as potential (co)sources of the found latitudinal metabolomic patterns. Furthermore, as the host plant species on which the mites were sampled in the field covaried with latitude (with *L. periclymenum* typically in the north), this could also have influenced our results. Yet, regression slopes barely differed between models including all populations (hence all host plant species) or models run for the subset of six populations collected on *L. periclymenum* only (see Appendix S8). This indicates that the latitudinal signal was independent of host plant identity.

This explorative study specifically examined whether range expansion might result in evolutionary changes in an organism's metabolism. Despite nonstressful common garden conditions, approximately 40% of the identified metabolites showed (epi)genetic differentiation among populations. The more dispersive northern mites exhibited lower concentrations of several essential and nonessential amino acids, suggesting a downregulation of certain pathways linked to protein synthesis. Although effects were subtle (but see earlier), our results clearly indicate that the metabolome of *T. urticae* underwent (epi)genetic changes during the species’ recent range expansion.

## Data accessibility

- Sample locations and life‐history trait values: included in appendix.
- Results of GC‐MS analysis: deposited in the Dryad repository http://dx.doi.org/10.5061/dryad.78cc8.
- Outcomes of statistical analyses: included in appendix.

## Conflict of Interest

None declared.

## Supporting information


**Appendix S1.** Overview of the field collection sites and their respective life‐history trait values.
**Appendix S2.** Overview of the 60 metabolites included in our spectral database.
**Appendix S3.** Correlation matrix.
**Appendix S4.** 3‐D score plots.
**Appendix S5.** Overview of the VIP scores resulting from the PLS‐DA.
**Appendix S6.** Overview of all linear regressions.
**Appendix S7.** Pathway maps.
**Appendix S8.** Regression for all populations versus for *L. periclymenum* only.
