# Supplementary material for: Metabolic adaptations in a range‐expanding arthropod
Source: Ecol Evol. 2016 Aug 23;6(18):6556–64. doi: 10.1002/ece3.2350 (PMC5058527; doi:10.1002/ece3.2350)
Supplement: Supplementary file 1 — Appendix S1. Overview of the field collection sites and their respective life‐history trait values. Appendix S2. Overview of the 60 metabolites included in our spectral database. Appendix S3. Correlation matrix. Appendix S4. 3‐D score plots. Appendix S5. Overview of the VIP scores resulting from the PLS‐DA. Appendix S6. Overview of all linear regressions. Appendix S7. Pathway maps. Appendix S8. Regression for all populations versus for L. periclymenum only. [file ECE3-6-6556-s001.docx]

**Appendix**

**A.1: Overview of the field collection sites and their respective life-history trait values**

**An overview of the localities where *T. urticae* was sampled in the field, together with population-level life-history trait values (daily fecundity (#eggs/day), lifetime fecundity (total #eggs), egg survival (%), longevity (#days), dispersal propensity (%) and sex ratio (#male/total)). These values, originating from a previous study (Van Petegem *et al.* 2016), were used as the independent values in our linear regressions. Field collection sites were located along a latitudinal gradient, spanning the coasts of Belgium (BEL), The Netherlands (NTL), Germany and Denmark (DEN). The denoted plant species (*Lonicera periclymenum*, *Euonymus europaeus*, *Sambucus nigra* and *Humulus lupulus*) are the species on which the mites were found and sampled.**

| code | collection site | city, country | Coordinates  (WGS 84) | plant species | daily  fecundity | lifetime  fecundity | egg  survival | longevity | dispersal  propensity | sex  ratio |
| --- | --- | --- | --- | --- | --- | --- | --- | --- | --- | --- |
| SKA | Flagbakkevej | Skagen, DEN | 57.72, 10.53 | *L. periclymenum* | 3.85 | 33.42 | 96.72 | 10.80 | 9.47 | 0.33 |
| SVI | Sletteåvej | Fjerritslev, DEN | 57.15, 9.33 | *L. periclymenum* | 5.19 | 29.33 | . | 6.33 | 41.41 | 0.32 |
| TVE | Hindingvej | Thisted, DEN | 57.04, 8.62 | *L. periclymenum* | 3.65 | 23.42 | 100.00 | 7.42 | 14.53 | 0.26 |
| BLA | V. Hennebysvej | Henne, DEN | 55.74, 8.22 | *L. periclymenum* | 3.72 | 40.92 | 100.00 | 11.00 | 39.47 | 0.29 |
| HED | picnic-place along 11/24 | Gredstedbro, DEN | 55.39, 8.74 | *L. periclymenum* | 3.48 | 29.17 | 96.88 | 9.60 | 31.45 | 0.22 |
| LAU | Schildhoek | Lauwersoog, NTL | 53.41, 6.22 | *E. europaeus* | 4.25 | 37.00 | 87.30 | 9.75 | 16.75 | 0.34 |
| CAS | Hoofdweg | Heemskerk, NTL | 52.53, 4.65 | *L. periclymenum* | 5.54 | 41.75 | 96.15 | 9.40 | 19.21 | 0.28 |
| KVS | Bosweg | Burgh-Haamstede, NTL | 51.68, 3.72 | *S. nigra* | 6.34 | 98.67 | 99.02 | 17.55 | 15.03 | 0.19 |
| ODK | Duinparklaan | Koksijde, BEL | 51.12, 2.68 | *H. lupulus* | 5.22 | 50.42 | 98.99 | 10.08 | 9.81 | 0.25 |

**A.2: Overview of the 60 metabolites included in our spectral database**

**A list of all 60 metabolites screened for in our single quadrupole GC-MS. Each of these metabolites can be characterised by several ions of different masses. We used the selective ion monitoring mode (SIM, see Waller *et al.* 2007) instead of the full scan mode. In this SIM mode, the MS instrument is set to only look for specific previously-known ion masses of interest rather than to screen for all masses over a wide range. The instrument can therefore be very specific for a particular compound of interest. Our original database contained spectral information for 85 primary metabolites from a range of plant and invertebrate models. After removing all metabolites specific to plant models and those (polyamines for instance) having concentrations lower than the detection limit of our equipment, only the 60 specific metabolites listed in the table below remained in our spectral database. Metabolites are classified according to six categories: amino acid, polyol, sugar, intermediate of the citric acid cycle, ‘other’ metabolite and metabolite not found in any of our samples.**

| category | metabolite |
| --- | --- |
| amino acid | Alanine |
| amino acid | Aspartic acid |
| amino acid | Glutamic acid |
| amino acid | Glycine |
| amino acid | Isoleucine |
| amino acid | Leucine |
| amino acid | Lysine |
| amino acid | Methionine |
| amino acid | Phenylalanine |
| amino acid | Proline |
| amino acid | Serine |
| amino acid | Threonine |
| amino acid | Tryptophan |
| amino acid | Tyrosine |
| amino acid | Valine |
| intermediate citric acid cycle | Citric acid |
| intermediate citric acid cycle | Fumaric acid |
| intermediate citric acid cycle | Malic acid |
| intermediate citric acid cycle | Succinic acid |
| polyol | Adonitol |
| polyol | Arabitol |
| polyol | Glycerol |
| polyol | Glycerol-3-phosphate |
| polyol | Inositol |
| polyol | Sorbitol |
| polyol | Xylitol |
| sugar | Fructose |
| sugar | Fructose-6-phosphate |
| sugar | Galactose |
| sugar | Glucose |
| sugar | Glucose-6-phosphate |
| sugar | Mannose |
| sugar | Ribose |
| other | Ethanolamine |
| other | GABA |
| other | Gluconolactone |
| other | Glyceric acid |
| other | Lactic acid |
| other | Ornithine |
| other | Phosphoric acid |
| other | Putrescine |
| other | Quinic acid |
| other | Spermidine |
| not found | Arabinose |
| not found | Asparagine |
| not found | Cadaverine |
| not found | Citrulline |
| not found | Cytosine |
| not found | Erythritol |
| not found | Galactitol |
| not found | Galacturonic acid |
| not found | Maltose |
| not found | Mannitol |
| not found | Octopamine |
| not found | Pipecolic acid |
| not found | Saccharose |
| not found | Spermine |
| not found | Trehalose |
| not found | Tyramine |
| not found | Xylose |

**A.3: correlation matrix**

**LAT (latitude), LIFE (lifetime fecundity), DAFE (daily fecundity), DISP (dispersal propensity), EGSU (egg survival), LONG (longevity), DIAP (diapause incidence) and SERA (sex ratio). All significant correlations (Bonferroni correction not implemented) are in bold. As only a selection of populations was included in the current study, the strength of the correlations between latitude and the shown life-history traits decreased relative to the correlations found in Van Petegem *et al.* (2016).**

| Pearson Correlation Coefficients | | | | | | | | |
| --- | --- | --- | --- | --- | --- | --- | --- | --- |
| Prob > \|r\| under H0: Rho=0 | | | | | | | | |
| Number of Observations | | | | | | | | |
|  | LAT | LIFE | DAFE | DISP | EGSU | LONG | DIAP | SERA |
| LAT | 1 | -0.67384 | -0.67452 | 0.38101 | 0.13882 | -0.52688 | 0.47464 | 0.43955 |
|  |  | **0.0466** | **0.0463** | 0.3117 | 0.743 | 0.145 | 0.1967 | 0.2365 |
|  | 9 | 9 | 9 | 9 | 8 | 9 | 9 | 9 |
| LIFE | -0.67384 | 1 | 0.75065 | -0.27469 | 0.18641 | 0.9203 | -0.337 | -0.5922 |
|  | **0.0466** |  | **0.0198** | 0.4744 | 0.6585 | **0.0004** | 0.3752 | 0.0929 |
|  | 9 | 9 | 9 | 9 | 8 | 9 | 9 | 9 |
| DAFE | -0.67452 | 0,75065 | 1 | -0.18302 | 0.06919 | 0.46919 | 0.13277 | -0.33554 |
|  | **0.0463** | **0.0198** |  | 0.6374 | 0.8707 | 0.2026 | 0.7335 | 0.3774 |
|  | 9 | 9 | 9 | 9 | 8 | 9 | 9 | 9 |
| DISP | 0.38101 | -0.27469 | -0.18302 | 1 | 0.14537 | -0.31841 | 0.66023 | 0.11912 |
|  | 0.3117 | 0.4744 | 0.6374 |  | 0.7313 | 0.4037 | 0.0529 | 0.7602 |
|  | 9 | 9 | 9 | 9 | 8 | 9 | 9 | 9 |
| EGSU | 0.13882 | 0.18641 | 0.06919 | 0.14537 | 1 | 0.16269 | 0.19129 | -0.59346 |
|  | 0.743 | 0.6585 | 0.8707 | 0.7313 |  | 0.7003 | 0.65 | 0.1209 |
|  | 8 | 8 | 8 | 8 | 8 | 8 | 8 | 8 |
| LONG | -0.52688 | 0.9203 | 0.46919 | -0.31841 | 0.16269 | 1 | -0.52876 | -0.54739 |
|  | 0.145 | **0.0004** | 0.2026 | 0.4037 | 0.7003 |  | 0.1433 | 0.1271 |
|  | 9 | 9 | 9 | 9 | 8 | 9 | 9 | 9 |
| DIAP | 0.47464 | -0.337 | 0.13277 | 0.66023 | 0.19129 | -0.52876 | 1 | 0.29697 |
|  | 0.1967 | 0.3752 | 0.7335 | 0.0529 | 0.65 | 0.1433 |  | 0.4377 |
|  | 9 | 9 | 9 | 9 | 8 | 9 | 9 | 9 |
| SERA | 0.43955 | -0.5922 | -0.33554 | 0.11912 | -0.59346 | -0.54739 | 0.29697 | 1 |
|  | 0.2365 | 0.0929 | 0.3774 | 0.7602 | 0.1209 | 0.1271 | 0.4377 |  |
|  | 9 | 9 | 9 | 9 | 8 | 9 | 9 | 9 |

**A.4: 3-D score plots**

**3-D score plots resulting from the multivariate analyses (PLS-DA) performed on our metabolomic data are given for latitude (A) and each of six life-history traits (daily fecundity (B), lifetime fecundity (C), egg survival (D), longevity (E), dispersal propensity (F) and sex ratio (G)). The mean metabolic phenotypes for each of the five replicates (four for SKA) of all nine populations (eight for egg survival) are represented by a dot and arranged in space according to their projections on three component axes. Each of these axes explains a certain amount (%) of the metabolic variation that is present in the dataset. More similar points (populations with a similar metabolome) are placed closer together.**


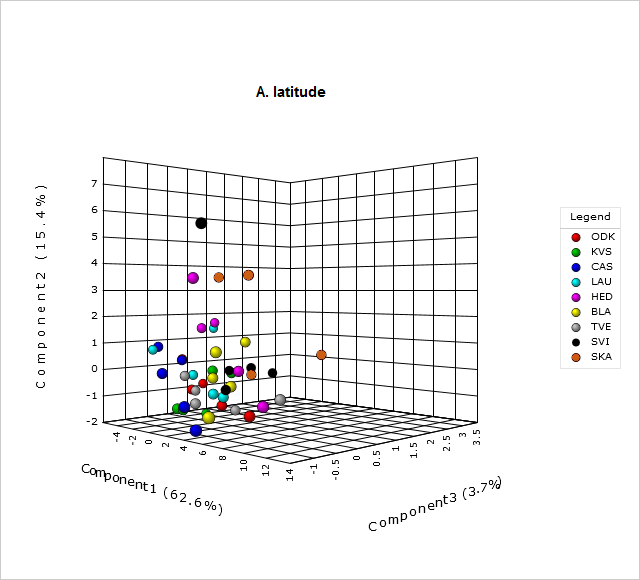


**
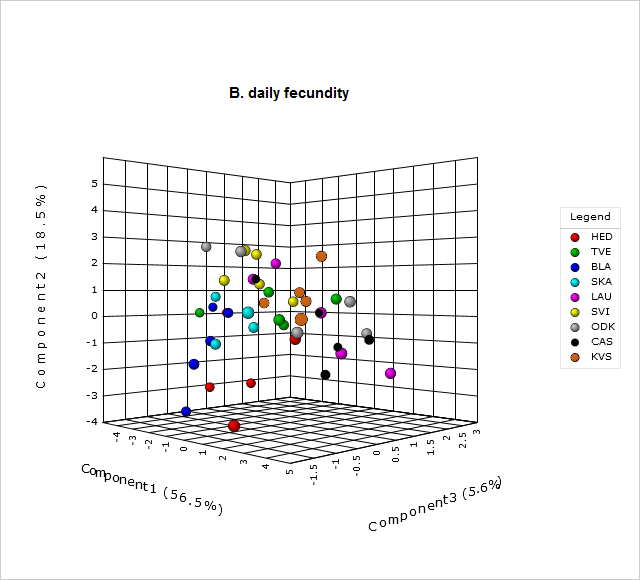
**

**
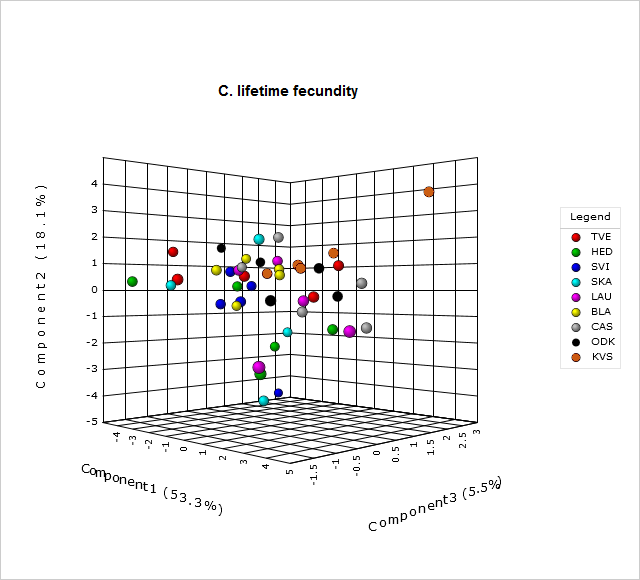
**

**
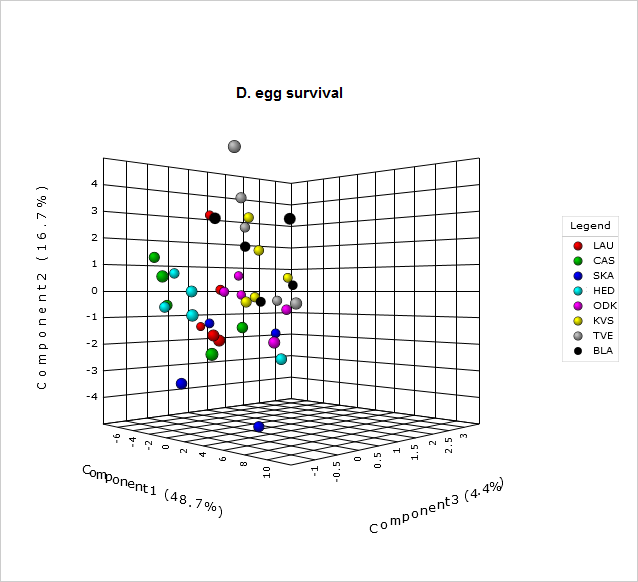
**

**
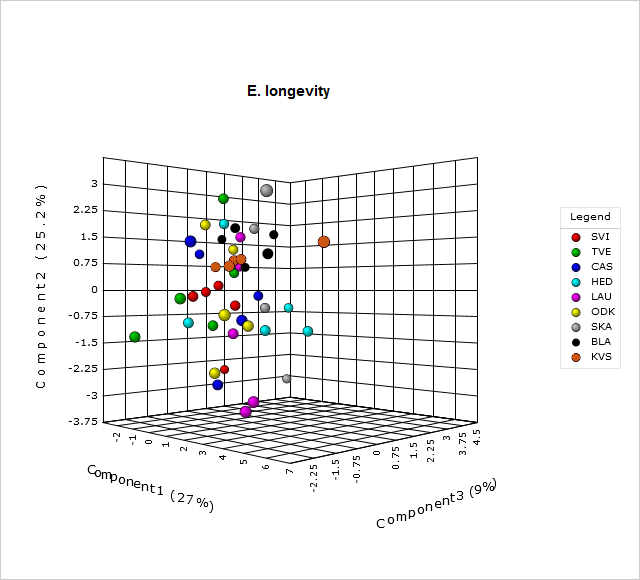
**

**
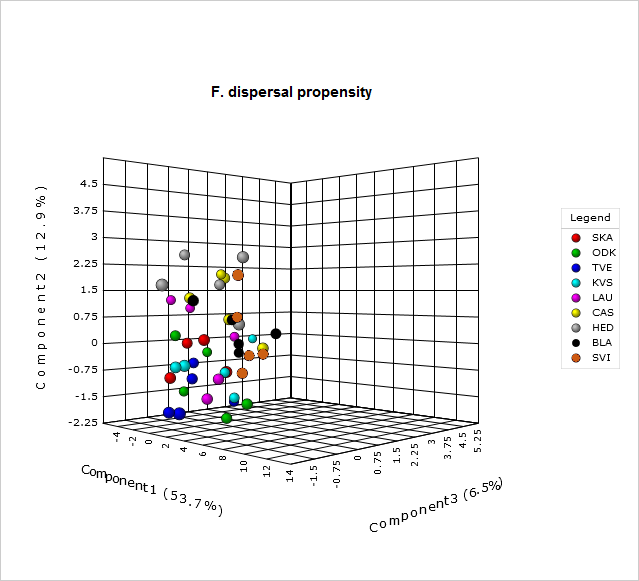
**

**
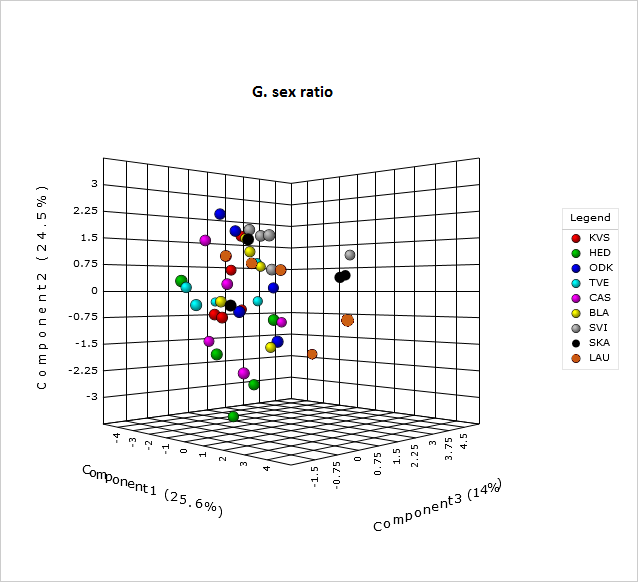
**

**A.5 overview of the VIP scores resulting from the PLS-DA**

**An overview off all metabolites with a VIP score of at least 1.2 (1.0 for egg survival because removing the metabolites with a score between 1.0 and 1.2 resulted in a decreased percentage of variation explained) for component 1 and/ or 2 in the PLS-DA performed for latitude (A) and each of six life-history traits (daily fecundity (B), lifetime fecundity (C), egg survival (D), longevity (E), dispersal propensity (F) and sex ratio(G)). Note that in the tables below, the VIP scores for the original dataset (containing all forty-three identified metabolites) are given. The order of the metabolites (from high to low VIP scores) in these tables can therefore deviate from the order in Fig. 3 (main text), as Fig. 3 is based on VIP scores of the final dataset (after removal of all non-explanatory metabolites).**

**A. latitude**

| metabolite | Comp. 1 | Comp. 2 |
| --- | --- | --- |
| Proline | 1.6583 | 1.0501 |
| Tryptophan | 1.5937 | 1.0289 |
| Inositol | 1.5831 | 1.0274 |
| Malic acid | 1.4947 | 0.9356 |
| Lysine | 1.4669 | 0.9290 |
| Valine | 1.4577 | 0.9261 |
| Isoleucine | 1.4219 | 0.9054 |
| Methionine | 1.4115 | 0.8879 |
| Glycine | 1.4090 | 0.8936 |
| Aspartic acid | 1.3881 | 0.8733 |
| Threonine | 1.3705 | 0.9118 |
| Alanine | 1.3572 | 0.8500 |
| Leucine | 1.3414 | 0.9084 |
| Quinic acid | 1.3313 | 1.6940 |
| Glucose-6-phosphate | 0.6854 | 2.0669 |
| Sorbitol | 1.0691 | 1.9186 |
| Fructose-6-phosphate | 0.4123 | 1.7967 |
| Ribose | 0.8471 | 1.2669 |

**B. daily fecundity**

| metabolite | Comp. 1 | Comp. 2 |
| --- | --- | --- |
| Glycerol | 1.7613 | 1.8109 |
| Phenylalanine | 1.6599 | 1.2292 |
| Sorbitol | 1.6526 | 1.4537 |
| Valine | 1.5163 | 1.1636 |
| Xylitol | 1.4767 | 1.2651 |
| Lysine | 1.4179 | 1.0710 |
| Isoleucine | 1.4127 | 1.1010 |
| Glutamic acid | 1.3973 | 0.9667 |
| Tyrosine | 1.3441 | 1.0950 |
| Leucine | 1.3261 | 1.1045 |
| Threonine | 1.2862 | 1.0977 |
| Inositol | 1.2444 | 0.9216 |
| Glycine | 1.2215 | 0.9496 |
| Tryptophan | 1.2095 | 0.9172 |

**C. lifetime fecundity**

| metabolite | Comp. 1 | Comp. 2 |
| --- | --- | --- |
| Lysine | 1.5753 | 0.8880 |
| Glycine | 1.5660 | 0.8728 |
| Tryptophan | 1.5312 | 0.8706 |
| Inositol | 1.5101 | 0.8566 |
| Valine | 1.4669 | 0.9187 |
| Proline | 1.4248 | 0.8628 |
| Alanine | 1.4119 | 0.7920 |
| Isoleucine | 1.3774 | 0.8873 |
| Malic acid | 1.3485 | 0.8655 |
| Phenylalanine | 1.3188 | 0.9131 |
| Leucine | 1.3161 | 0.9319 |
| Lactic acid | 1.2638 | 1.3055 |
| Glucose-6-phosphate | 1.2298 | 1.7909 |
| Quinic acid | 1.2139 | 0.9298 |
| Fructose-6-phosphate | 0.9630 | 1.6380 |

**D. egg survival**

| metabolite | Comp. 1 | Comp. 2 |
| --- | --- | --- |
| Glutamic acid | 1.7874 | 1.3641 |
| Alanine | 1.6795 | 1.1028 |
| Tyrosine | 1.4029 | 0.9337 |
| Phenylalanine | 1.3828 | 0.9234 |
| Fructose-6-phosphate | 1.3581 | 0.8561 |
| Glucose-6-phosphate | 1.3492 | 0.8500 |
| Methionine | 1.2596 | 0.8576 |
| Xylitol | 1.2585 | 1.0527 |
| Glycerol-3-phosphate | 1.2193 | 0.7804 |
| Citric acid | 1.2164 | 0.8394 |
| Serine | 1.1977 | 0.9293 |
| GABA | 1.1951 | 2.2339 |
| Gluconolactone | 1.1857 | 0.8810 |
| Adonitol | 1.1823 | 0.8112 |
| Succinic acid | 1.1628 | 1.0936 |
| Leucine | 1.1035 | 0.9157 |
| Threonine | 1.0640 | 0.9255 |
| Valine | 1.0216 | 0.8728 |
| Ornithine | 1.0141 | 0.6404 |
| Ethanolamine | 0.8772 | 1.9297 |
| Glycerol | 0.5981 | 1.7154 |
| Fumaric acid | 0.0152 | 1.5159 |
| Glutamic acid | 1.7874 | 1.3641 |
| Glyceric acid | 2.1181 | 1.3224 |

**E. longevity**

| metabolite | Comp. 1 | Comp. 2 |
| --- | --- | --- |
| Glucose | 2.1463 | 1.4658 |
| Succinic acid | 1.9802 | 1.3826 |
| Sorbitol | 1.9329 | 1.3478 |
| Citric acid | 1.6265 | 1.5863 |
| Glycine | 1.4818 | 1.1018 |
| Galactose | 1.4447 | 1.1659 |
| Lysine | 1.4091 | 1.0542 |
| Lactic acid | 1.3965 | 1.0108 |
| Fructose-6-phosphate | 1.3276 | 1.4043 |
| Glucose-6-phosphate | 1.3168 | 1.3696 |
| Phosphoric acid | 1.2220 | 1.0486 |

**F. dispersal propensity**

| metabolite | Comp. 1 | Comp. 2 |
| --- | --- | --- |
| Putrescine | 1.9187 | 1.2144 |
| Phosphoric acid | 1.7678 | 1.0416 |
| Lysine | 1.7503 | 1.0280 |
| Methionine | 1.7343 | 1.0303 |
| Xylitol | 1.7230 | 1.9394 |
| Phenylalanine | 1.5574 | 0.9821 |
| Fructose | 1.4964 | 0.9235 |
| Tryptophan | 1.2684 | 0.7950 |
| Glycerol-3-phosphate | 1.2602 | 0.7514 |
| Leucine | 1.2553 | 0.9363 |
| Inositol | 1.2549 | 0.7824 |
| Glucose-6-phosphate | 0.6820 | 1.4379 |
| Mannose | 0.2110 | 1.3947 |

**G. sex ratio**

| metabolite | Comp. 1 | Comp. 2 |
| --- | --- | --- |
| GABA | 2.7316 | 2.5800 |
| Succinic acid | 2.5829 | 2.4256 |
| Glutamic acid | 2.0087 | 1.8893 |
| Glucose-6-phosphate | 1.7334 | 1.6527 |
| Sorbitol | 1.5872 | 1.4965 |
| Citric acid | 1.4820 | 1.4001 |
| Fructose-6-phosphate | 1.4541 | 1.3954 |
| Xylitol | 1.2478 | 1.3554 |
| Ethanolamine | 1.2072 | 1.3270 |
| Fructose | 0.9418 | 0.9842* |

**** After removal of the least explanatory metabolites in the dataset, the VIP score of fructose changed to >1.2. Fructose was therefore retained for further analysis.***

**A.6 overview of all linear regressions**

**An overview of all linear regressions according to latitude (A) and each of the six life-history traits (daily fecundity (B), lifetime fecundity (C), egg survival (D), longevity (E), dispersal propensity (F) and sex ratio(G)) known to covary with latitude in the study species (see Van Petegem *et al.* 2016). The direction of change (correlation) is each time given, together with the F- and *p-*value of the linear regression. The degrees of freedom used in determining the F-value (Num DF and Den DF) are also shown.**

**A. latitude**

| metabolite | correlation | Num DF | Den DF | F | p |
| --- | --- | --- | --- | --- | --- |
| Proline | negative | 1 | 7 | 9.85 | **0.0164** |
| Tryptophan | negative | 1 | 7 | 6.48 | **0.0383** |
| Inositol | negative | 1 | 7 | 6.43 | **0.0389** |
| Aspartic acid | negative | 1 | 7 | 6.62 | **0.0369** |
| Glycine | negative | 1 | 7 | 7.19 | **0.0315** |
| Malic acid | negative | 1 | 7 | 6.59 | **0.0372** |
| Valine | negative | 1 | 7 | 8.15 | **0.0245** |
| Lysine | negative | 1 | 7 | 3.77 | 0.0932 |
| Alanine | negative | 1 | 7 | 4.16 | 0.0808 |
| Methionine | negative | 1 | 7 | 3.06 | 0.1238 |
| Isoleucine | negative | 1 | 7 | 8.81 | **0.0209** |
| Quinic acid | positive | 1 | 7 | 5.59 | **0.0500** |
| Threonine | negative | 1 | 7 | 7.35 | **0.0301** |
| Leucine | negative | 1 | 7 | 6.71 | **0.0359** |
| Sorbitol | positive | 1 | 7 | 1.13 | 0.3221 |
| Glucose-6-phosphate | positive | 1 | 7 | 1.00 | 0.3511 |
| Fructose-6-phosphate | positive | 1 | 7 | 0.34 | 0.5789 |

**B. daily fecundity**

| metabolite | correlation | Num DF | Den DF | F | p |
| --- | --- | --- | --- | --- | --- |
| Glycerol | negative | 1 | 7 | 1.91 | 0.2096 |
| Phenylalanine | positive | 1 | 7 | 1.87 | 0.2135 |
| Glutamic acid | positive | 1 | 7 | 0.47 | 0.5193 |
| Sorbitol | negative | 1 | 7 | 3.37 | 0.0947 |
| Valine | positive | 1 | 7 | 2.41 | 0.1648 |
| Isoleucine | positive | 1 | 7 | 2.46 | 0.1606 |
| Lysine | positive | 1 | 7 | 1.34 | 0.2588 |
| Tyrosine | positive | 1 | 7 | 1.31 | 0.2897 |
| Leucine | positive | 1 | 7 | 1.54 | 0.2553 |
| Threonine | positive | 1 | 7 | 1.83 | 0.2182 |
| Xylitol | negative | 1 | 7 | 0.46 | 0.5193 |
| Glycine | positive | 1 | 7 | 1.51 | 0.2587 |
| Inositol | positive | 1 | 7 | 0.71 | 0.4284 |
| Tryptophan | positive | 1 | 7 | 0.62 | 0.4584 |

**C. lifetime fecundity**

| metabolite | correlation | Num DF | Den DF | F | p |
| --- | --- | --- | --- | --- | --- |
| Lysine | positive | 1 | 7 | 2.57 | 0.1531 |
| Glycine | positive | 1 | 7 | 3.86 | 0.0903 |
| Tryptophan | positive | 1 | 7 | 0.60 | 0.4629 |
| Inositol | positive | 1 | 7 | 0.61 | 0.4592 |
| Valine | positive | 1 | 7 | 1.61 | 0.2455 |
| Proline | positive | 1 | 7 | 0.36 | 0.5665 |
| Alanine | positive | 1 | 7 | 0.15 | 0.7089 |
| Isoleucine | positive | 1 | 7 | 1.47 | 0.2653 |
| Malic acid | positive | 1 | 7 | 1.24 | 0.3026 |
| Phenylalanine | positive | 1 | 7 | 0.70 | 0.4317 |
| Leucine | positive | 1 | 7 | 0.91 | 0.3717 |
| Lactatic acid | positive | 1 | 7 | 0.22 | 0.6503 |
| Glucose-6-phosphate | negative | 1 | 7 | 2.38 | 0.1671 |
| Quinic acid | negative | 1 | 7 | 0.58 | 0.4716 |
| Fructose-6-phosphate | negative | 1 | 7 | 2.15 | 0.1862 |
| Glycerol-3-phosphate | negative | 1 | 7 | 1.07 | 0.3363 |

**D. egg survival**

| metabolite | correlation | Num DF | Den DF | F | p |
| --- | --- | --- | --- | --- | --- |
| Glutamic acid | negative | 1 | 6 | 3.79 | 0.0994 |
| Alanine | negative | 1 | 6 | 4.40 | 0.0808 |
| Tyrosine | negative | 1 | 6 | 5.24 | 0.0621 |
| Phenylalanine | negative | 1 | 6 | 2.70 | 0.1516 |
| Fructose-6-phosphate | negative | 1 | 6 | 4.55 | 0.0770 |
| Glucose-6-phosphate | negative | 1 | 6 | 4.66 | 0.0742 |
| Methionine | negative | 1 | 6 | 1.84 | 0.2238 |
| Xylitol | negative | 1 | 6 | 0.37 | 0.5649 |
| Succinic acid | negative | 1 | 6 | 3.02 | 0.1327 |
| Glycerol-3-phosphate | negative | 1 | 6 | 4.70 | 0.0733 |
| Citric acid | negative | 1 | 6 | 4.61 | 0.0754 |
| Serine | negative | 1 | 6 | 5.03 | 0.0661 |
| GABA | positive | 1 | 6 | 0.38 | 0.5580 |
| Adonitol | negative | 1 | 6 | 9.07 | **0.0237** |
| Gluconolactone | negative | 1 | 6 | 0.60 | 0.4697 |
| Leucine | negative | 1 | 6 | 3.16 | 0.1260 |
| Threonine | negative | 1 | 6 | 2.85 | 0.1425 |
| Valine | negative | 1 | 6 | 2.23 | 0.1860 |
| Ornithine | negative | 1 | 6 | 2.68 | 0.1525 |
| Ethanolamine | negative | 1 | 6 | 0.00 | 0.9891 |
| Glycerol | positive | 1 | 6 | 0.03 | 0.8651 |
| Malic acid | negative | 1 | 6 | 2.22 | 0.1871 |
| Glyceric acid | negative | 1 | 6 | 0.94 | 0.3698 |
| Fumaric acid | negative | 1 | 6 | 0.44 | 0.5305 |

**E. longevity**

| metabolite | correlation | Num DF | Den DF | F | p |
| --- | --- | --- | --- | --- | --- |
| Glucose | positive | 1 | 7 | 0.52 | 0.4955 |
| Succinic acid | negative | 1 | 7 | 0.00 | 0.9482 |
| Sorbitol | positive | 1 | 7 | 0.05 | 0.8306 |
| Citric acid | negative | 1 | 7 | 2.14 | 0.1870 |
| Glycine | positive | 1 | 7 | 2.83 | 0.1365 |
| Galactose | positive | 1 | 7 | 1.13 | 0.3236 |
| Lysine | positive | 1 | 7 | 1.65 | 0.2404 |
| Lactic acid | positive | 1 | 7 | 0.89 | 0.3777 |
| Fructose-6-phosphate | negative | 1 | 7 | 1.16 | 0.3174 |
| Glucose-6-phosphate | negative | 1 | 7 | 1.16 | 0.3172 |
| Phosphoric acid | positive | 1 | 7 | 0.97 | 0.3573 |
| Ornithine | positive | 1 | 7 | 0.79 | 0.4023 |
| Ribose | positive | 1 | 7 | 0.23 | 0.6430 |
| Xylitol | negative | 1 | 7 | 0.07 | 0.7935 |
| Ethanolamine | positive | 1 | 7 | 1.09 | 0.3310 |
| Glycerol | positive | 1 | 7 | 0.06 | 0.8184 |
| Glutamic acid | positive | 1 | 7 | 0.18 | 0.6826 |
| Mannose | positive | 1 | 7 | 0.88 | 0.3804 |
| Glycerol-3-phosphate | negative | 1 | 7 | 0.61 | 0.4607 |
| Methionine | positive | 1 | 7 | 0.05 | 0.8332 |
| Arabitol | positive | 1 | 7 | 0.15 | 0.7066 |
| Serine | positive | 1 | 7 | 0.15 | 0.7117 |
| Adonitol | positive | 1 | 7 | 0.00 | 0.9879 |

**F. dispersal propensity**

| metabolite | correlation | Num DF | Den DF | F | p |
| --- | --- | --- | --- | --- | --- |
| Putrescine | negative | 1 | 7 | 8.64 | **0.0218** |
| Phosphoric acid | negative | 1 | 7 | 7.33 | **0.0303** |
| Lysine | negative | 1 | 7 | 6.38 | **0.0394** |
| Methionine | negative | 1 | 7 | 8.16 | **0.0245** |
| Xylitol | positive | 1 | 7 | 0.66 | 0.4436 |
| Phenylalanine | negative | 1 | 7 | 9.05 | **0.0197** |
| Fructose | negative | 1 | 7 | 7.29 | **0.0307** |
| Tryptophan | negative | 1 | 7 | 5.10 | 0.0585 |
| Glycerol-3-phosphate | negative | 1 | 7 | 3.67 | 0.0969 |
| Leucine | negative | 1 | 7 | 8.14 | **0.0246** |
| Inositol | negative | 1 | 7 | 4.94 | 0.0615 |
| Glucose-6-phosphate | positive | 1 | 7 | 0.37 | 0.5608 |
| Mannose | negative | 1 | 7 | 0.02 | 0.9013 |

**G. sex ratio**

| metabolite | correlation | Num DF | Den DF | F | p |
| --- | --- | --- | --- | --- | --- |
| GABA | negative | 1 | 7 | 3.26 | 0.1141 |
| Succinic acid | positive | 1 | 7 | 3.49 | 0.1038 |
| Glutamic acid | positive | 1 | 7 | 0.91 | 0.3716 |
| Glucose-6-phosphate | positive | 1 | 7 | 2.60 | 0.1511 |
| Sorbitol | positive | 1 | 7 | 0.83 | 0.3932 |
| Citric acid | positive | 1 | 7 | 1.82 | 0.2193 |
| Fructose-6-phosphate | positive | 1 | 7 | 1.91 | 0.2089 |
| Xylitol | negative | 1 | 7 | 0.26 | 0.6281 |
| Ethanolamine | negative | 1 | 7 | 1.20 | 0.3091 |
| Fructose | negative | 1 | 7 | 0.70 | 0.4294 |

**A.7 pathway maps**

**Pathway maps of the two pathways in which some of the metabolites identified in the GC-MS analysis (visualised with either red or blue rectangles) play an important role. In the aminoacyl-tRNA biosynthesis pathway, the metabolites in the red rectangles correlate negatively with latitude and the blue ones correlate negatively with dispersal propensity. In the valine, leucine and isoleucine biosynthesis pathway, the metabolites in the red rectangles correlate negatively with latitude. The maps were used from KEGG (Kyoto Encyclopedia of Genes and Genomes) (Kanehisa *et al.* 2015).**

**
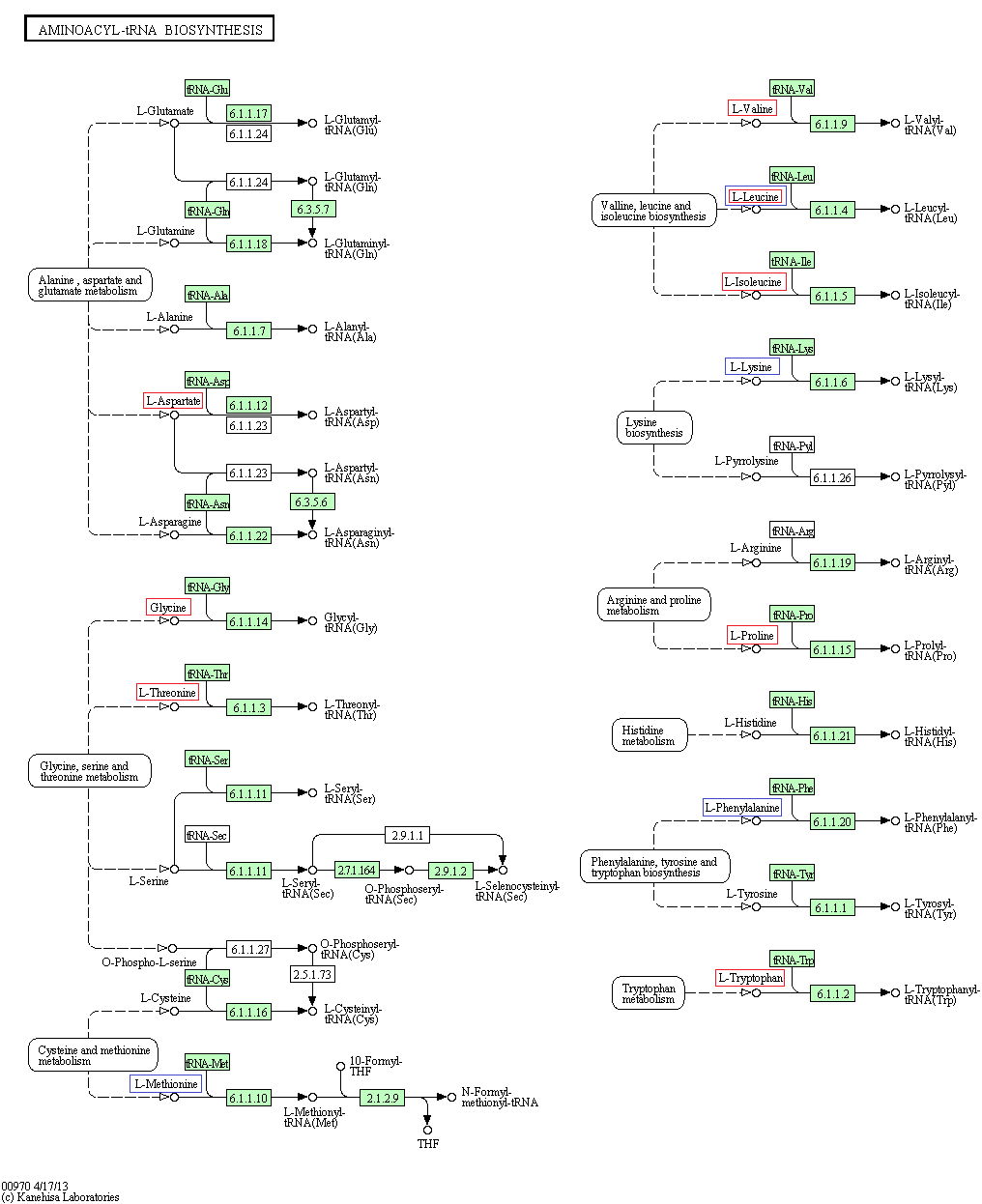
**

**
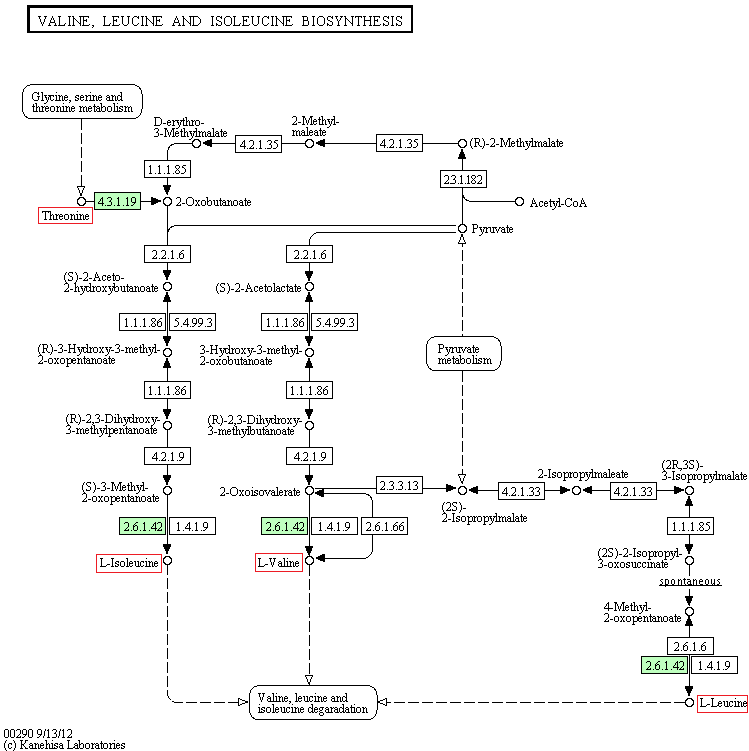
**

**A.8 regression for all populations *versus* for *L. periclymenum* only**

**For those metabolites that gave significant results (see appendix A.6) for latitude or a specific life-history trait (daily fecundity, lifetime fecundity, egg survival, longevity, dispersal propensity or sex ratio) in the regressions run for all nine populations (hence for *L. periclymenum*, *Euonymus europaeus*, *Sambucus nigra* and *Humulus lupulus*), we repeated these regressions but then for *L. periclymenum* only (hence for only six populations –see appendix A.1). The table gives the slopes and p*-*values of both the regressions run for all populations (slope_all and p_all) and those run for *L. periclymenum* only (slope_*per* and p_*per*).**

| trait | metabolite | slope_*per* | slope_all | p_*per* | p_all |
| --- | --- | --- | --- | --- | --- |
| latitude | Proline | -0.04354 | -0.02733 | 0.0205 | 0.0164 |
| latitude | Tryptophan | -0.05170 | -0.04474 | 0.1766 | 0.0383 |
| latitude | Inositol | -0.05628 | -0.04890 | 0.1880 | 0.0389 |
| latitude | Aspartic acid | -0.01956 | -0.02840 | 0.2757 | 0.0369 |
| latitude | Glycine | -0.00681 | -0.03254 | 0.6500 | 0.0315 |
| latitude | Malic acid | -0.00703 | -0.03858 | 0.6992 | 0.0372 |
| latitude | Valine | -0.02914 | -0.02727 | 0.1726 | 0.0245 |
| latitude | Isoleucine | -0.03947 | -0.02997 | 0.0947 | 0.0209 |
| latitude | Quinic acid | 0.02094 | 0.01594 | 0.2119 | 0.0500 |
| latitude | Threonine | -0.02807 | -0.02700 | 0.2046 | 0.0301 |
| latitude | Leucine | -0.01727 | -0.02270 | 0.3466 | 0.0359 |
| egg survival | Adonitol | -0.00651 | -0.00258 | 0.0751 | 0.0237 |
| dispersal propensity | Putrescine | -0.00092 | -0.00108 | 0.0854 | 0.0218 |
| dispersal propensity | Phosphoric acid | -0.10219 | -0.13336 | 0.0492 | 0.0303 |
| dispersal propensity | Lysine | -0.00277 | -0.00553 | 0.0942 | 0.0394 |
| dispersal propensity | Methionine | -0.00793 | -0.01081 | 0.1461 | 0.0245 |
| dispersal propensity | Phenylalanine | -0.01652 | -0.02201 | 0.1208 | 0.0197 |
| dispersal propensity | Fructose | -0.00266 | -0.00221 | 0.0744 | 0.0307 |
| dispersal propensity | Leucine | -0.03211 | -0.04462 | 0.1224 | 0.0246 |

**References**

Kanehisa, M., Sato, Y., Kawashima, M., Furumichi, M. & Tanabe, M. (2015) KEGG as a reference resource for gene and protein annotation. *Nucleic Acids Research*.

Van Petegem, K., Boeye, J., Stoks, R. & Bonte, D. (2016) Spatial selection and local adaptation jointly shape life-history evolution during range expansion. *American Naturalist*, **in press**. Doi BioRxiv: http://dx.doi.org/10.1101/031922

Waller, M., Whitney, R., Taylor, M., Miyagawa, H., Matsuda, K. & Nakagawa, K. (2007) Multiresidue pesticides analysis using synchronous Scan/SIM mode GC-MS. *Lc Gc North America***,** 39-39.
